# Comparison of pectoralis major muscle satellite cell assay methods: an opinion paper

**DOI:** 10.3389/fphys.2024.1370570

**Published:** 2024-02-14

**Authors:** Sandra G. Velleman

**Affiliations:** Department of Animal Sciences, The Ohio State University, Columbus, OH, United States

**Keywords:** breast muscle, chicken, pectoralis major muscle, satellite cells, turkey

## Introduction

The identification of satellite cells by electron microscopy juxtaposed to myofibers was originally reported by Mauro (1961). Since the initial discovery in 1961, numerous studies have been conducted determining the essential role of satellite cells in posthatch skeletal muscle growth, and the repair and regeneration of myofibers following injury. As first shown by Smith (1963), muscle fiber number is determined embryonically, and all posthatch muscle fiber growth is through increased nuclear content in the myofibers through the fusion of satellite cells with existing muscle fibers. To repair and regenerate damaged myofibers, satellite cells are activated to proliferate and differentiate to reestablish a myofiber like the originating fiber. To study the complexity of satellite cell biological function, *in vitro* cell culture methods have been developed. The implementation of satellite cell culture studies has increased our understanding of the mechanistic role of satellite cells in regulating posthatch muscle growth (McFarland et al., 1991; Velleman et al., 2000), the regeneration of myofibers (Velleman et al., 2018), and the involvement of satellite cells in myopathies like Wooden Breast (Velleman, 2023).

Satellite cells are not a homogeneous population of cells. Frequently in the literature, it is implied that satellite cells are a uniform population of cells while the existence of multiple populations and the biological significance of their heterogeneity is often overlooked. The heterogeneity of satellite cells is reviewed in Velleman (2022). The first report of satellite cell heterogeneity was based on satellite cell proliferation being age-dependent (Schultz and Lipton, 1982). Subsequent to this initial finding of satellite cell age related changes in biological function, other studies have shown heterogeneity in satellite cells from different muscle fiber types expressing the genes of the originating fiber type (Feldman and Stockdale, 1991), satellite cells isolated from the same myofiber having varying rates of proliferation and differentiation (McFarland et al., 1995; Schultz, 1996), and satellite cell populations in the pectoralis major muscle being affected by growth selection (Xu et al., 2021; 2023). The McFarland et al. (1995) study showed heterogeneity within a single fiber type muscle with isolating both fast and slow growing satellite cells from a single pectoralis major muscle from a 6 week old tom turkey. The fastest growing satellite cells reached 65% confluency during proliferation in 17 days compared to 30 days for the slower growing cells.

For the commercial poultry industry, these differences in satellite cell growth and regeneration potential becomes important in determining meat quality especially for the breast muscle where costly downgrades have been occurring due to myopathies like Wooden Breast and Spaghetti Meat. Changes in satellite cell functional activity will affect their biological activity, including but not limited to, cell migration, growth factor responsiveness, and proliferation and differentiation. Interestingly, selection for rapid breast muscle growth in broilers and turkeys has differentially altered the satellite cells. In broilers, satellite cell proliferation and differentiation has been diminished in growth selected birds (Xu and Velleman, 2023). In contrast to the broilers, selection for rapid growth and muscling in meat-type turkeys has increased satellite cell proliferation and differentiation (Xu et al., 2021). The decrease in broiler satellite cell proliferation and differentiation may be associated with myopathies like Wooden Breast requiring the repair and regeneration of myofibers which is compromised with the condition (Velleman et al., 2018).

## Methods used to study avian satellite cells

The purpose of this section is not to provide detailed satellite cell isolation methods as they have been published (McFarland et al., 1988; McFarland, 2000), but to provide the author’s opinion as to when specific methodological approaches should be used. Despite satellite cells being a critical cell population, they comprise a small percentage of the posthatch adult cell number of muscle. At the time of hatch, satellite cells are 30% of the total number of nuclei in muscle. After muscle growth is completed, satellite cells are only 1%–5% of the total myonuclei (Hawke and Garry, 2001) with decreased proliferation and differentiation potential (Velleman et al., 2010).

Satellite cells were first isolated by Bischoff in 1974 from adult rat skeletal muscle (Bischoff, 1974). Viable primary satellite cells were liberated from the muscle tissue using enzymatic treatments like pronase or trypsin. The cells were further separated from tissue debris with differential centrifugation. This approach in general is still used with some modifications to isolate primary avian satellite cells for cell culture.

### Direct culturing of isolated primary satellite cells or freezing primaries in liquid nitrogen for multiple experimental usage

Many avian satellite cell studies have used a direct isolation of the primary cells with their immediate culture for experimentation as illustrated by Halevy and Lerman (1993). After the cells are liberated and debris removed, they are preplated for 2 h on uncoated cell culture wells to remove fibroblasts. After the preplating, the cells will be seeded onto 0.1% gelatin coated cell culture wells for experimentation on proliferation or differentiation, for example. As a note, the preplating step is needed with chicken satellite cells to remove fibroblasts which will overtake a muscle type cells in terms of their growth.

Direct culture of primary satellite cells allows the comparison of birds raised independently which does add power to the results. However, each experiment will require the isolation of new cells. It is difficult and costly to maintain a continuous population of birds with having the exact ages needed. Satellite cell activity changes greatly with age (Velleman et al., 2010). Variables in the rearing of the birds will alter satellite cell activity. Both extrinsic temperature (Xu et al., 2021; Xu and Velleman, 2023) and nutrition (Velleman et al., 2019) have been shown to affect satellite cell proliferation and differentiation. Thus, a primary cell culture approach with immediate experimentation is a challenging strategy for maintaining a continuous population of birds at the same developmental stage and with rearing conditions being consistent between different groups of birds.

McFarland et al. (1988) developed a freeze down and longterm storage in liquid nitrogen procedure for primary satellite cells. Thus, providing ample satellite cells for multiple experiments. The development of this procedure eliminates the expense and challenges in raising continuous populations of birds and variables in the birds affecting satellite cell biological function. Numerous experimental manipulations can be done in a significantly reduced time frame. In brief, following exsanguination of the birds, the pectoralis major muscle is removed using sterile procedures. The number of birds used varies between 10 and 20 depending on their age. The muscles are ground in a sterile meat grinder, finely minced and digested with pronase to liberate the satellite cells. To remove tissue debris, the suspensions are differentially centrifuged and then filtered through nylon cloth membranes with a pore size from 500 to 53 µm. For chickens, the cells are preplated in Dulbecco’s Modified Eagle Medium with 10% horse serum in uncoated cell culture wells for 2 h to remove fibroblasts in a 38.5°C 95% air/5% CO_2_ incubator. After the 2 h incubation, the cells are filtered through a 23 µm nylon cloth filter. The cells are now ready to freeze down and store in liquid nitrogen as primary cell isolates. The cells are stored in liquid nitrogen in Dulbecco’s Modified Eagle Medium containing 20% horse serum and 10% dimethylsulfoxide. The primary isolates can be expanded to a fifth or sixth passage for use which provides a plentiful supply of cells for experimentation. In my own research, satellite cells from a third or fourth passage are usually used in experiments. In general, a cryovial with approximately 500,000 cells will generate 20 to 30 vials with 500,000 cells per vial when expanded. The number of vials can vary based upon cellular growth characteristics of the isolated satellite cells which will be influenced by age and genetic line.

### Clonal isolation of satellite cells to study the biology of individual satellite cells isolated from the same muscle

Satellite cell heterogeneity exists in a single fiber type muscle like the turkey and chicken pectoralis major muscle which contains all type IIb fibers or even within an individual myofiber. To investigate the biological properties of satellite cell population differences requires the isolation and expansion of individual satellite cells. McFarland (2000) published a procedure for clonal expansion of an individual satellite cell. The McFarland (2000) procedure used robotic microscope assisted cell isolation and transfer. In brief, from a primary cell culture plate, individual cells are robotically transferred to a well in a 96 well cell culture plate. The cells proliferate until 65% confluency and then are transferred to larger diameter plates. When sufficiently expanded, the clonal satellite cells will be frozen down and stored in liquid nitrogen for experimental use. Using clonal isolation of satellite cells, McFarland et al. (1995) isolated 73 different satellite cells from the pectoralis major muscle of a single tom turkey that was 6 weeks of age. The clones were expanded to use in proliferation, differentiation, and growth factor responsiveness assays. A wide range of growth and differentiation differences were found from the satellite cell populations growing rapidly to those whose growth was delayed. For example, the slowest growing satellite cell took 30 days to reach 65% confluency in its growth whereas the fastest one only took 17 days. Differentiation was affected similarly to proliferation. Learning the growth and differentiation potential of different satellite cell populations is important in predicting muscle mass accretion. Slower growing and differentiating cells will have reduced muscle growth and could affect the morphological structure of the pectoralis major muscle. Differences in growth are supported by the altered growth factor responsiveness affecting both proliferation and differentiation. Meat quality is, in part, dependent upon the morphological structure of the breast muscle.

Fluorescence activated cell sorting or flow cytometry can be used to identify different populations of satellite cells by quantifying cell surface markers that may be differentially expressed. This is a particularly useful method to determine how genetic selection, age, or even extrinsic variables like temperature or nutrition have altered the presence of specific satellite cell types. Flow cytometry requires specific antibodies for the cell surface markers being quantified. Xu et al. (2023) quantified the effect of growth selection and temperature effects of syndecan-4 (SDC4) and CD44 populations of pectoralis major satellite cells isolated from a turkey slower-growing historic Randombred Control Line 2 (RBC2) and a modern commercial meat-type turkey (NC) line. Syndecan-4 is a transmembrane heparan sulfate proteoglycan that regulates proliferation (Velleman et al., 2007) and migration (Shin et al., 2013) of turkey satellite cells. CD44 is a transmembrane protein receptor whose extracellular domain binds to hyaluronic acid (Aruffo et al., 1990) and osteopontin (Weber et al., 1996). When CD44 binds to hyaluronic acid, it promotes proliferation, migration and myotube formation (Mylona et al., 2006; Leng et al., 2019). In brief, the satellite cells from both lines of turkeys were treated with hot (43°C) and cold (33°C) thermal stress for 72 h of proliferation followed by 48 h of differentiation. The NC line satellite cells had a lower proportion of SDC4 positive and CD44 negative (SDC4^+^CD44^−^) cells and more negative SDC4 cells and CD44 positive (SDC4^−^CD44^+^) cells compared to the RBC2 line at the control temperature (38°C) at both 72 h of proliferation and 48 h of differentiation. At 72 h of proliferation, the proportion of SDC4^+^CD44^−^ cells decreased at 43°C and increased with cold stress (33°C) compared to the control temperature (38°C) in both lines. The proportion of SDC4^−^CD44^+^ cells increased with heat stress and decreased with cold stress. In general, the expression of SDC4 and CD44 in the NC satellite cells had a greater response to both hot and cold thermal stress compared to the RBC2 cells. With more temperature extremes predicted due to climate change, it will be of value to determine the effect of growth selection on the expression of specific satellite cell populations and the impact on breast meat quality.

The procedures discussed are all acceptable to study the biological properties of avian pectoralis major satellite cells. What methods are selected must be tailored to resources available and the specific experimental objectives.
